# A Preliminary Study of the Immediate Effects of Spherical-Grip and Palmar-Support Handwriting Assistive Devices on Handwriting Kinematics During Numeric Copying in Children with Disabilities

**DOI:** 10.3390/children13101385

**Published:** 2026-10-12

**Authors:** Seong Ho Yun, Seon-Deok Eun

**Affiliations:** 1 Assistive Technology Research Team for Independent Living, National Rehabilitation Center, Seoul 01022, Republic of Korea; yshpt2107@naver.com; 2 Department of Healthcare and Public Health Research, National Rehabilitation Center, Seoul 01022, Republic of Korea

**Keywords:** handwriting, handwriting assistive device, children with disabilities

## Abstract

**Highlights:**

**What are the main findings?**

- In this exploratory preliminary study, the spherical-grip handwriting assistive device (AD) was associated with shorter writing duration and pen-tip path length compared with the no-AD condition.
- No statistically significant differences were detected between the palmar-support and no-AD conditions in the measured handwriting kinematic variables.

**What are the implications of the main findings?**

- The effects of handwriting ADs may vary according to how each device modifies the grasp and support interface.
- Quantitative handwriting kinematics may provide objective information for the evaluation of handwriting ADs in children with disabilities.

**Abstract:**

**Background**: Children with disabilities may experience difficulty with handwriting due to motor impairments that limit their ability to grasp and manipulate a pen. **Objectives**: This preliminary exploratory study investigated the effects of handwriting assistive devices (ADs) on handwriting kinematics in children with disabilities. **Methods**: A quasi-experimental within-subject repeated-measures design was used. Seven children with disabilities affecting hand function participated in this study. The children performed a numeric copying task under three conditions: no assistive device (no-AD), spherical-grip AD, and palmar-support AD. Handwriting kinematics were measured and compared across the three conditions. **Results**: The spherical-grip AD was associated with shorter writing duration and path length than the no-AD condition. No other statistically significant differences were observed among the three conditions. **Applications**: Our findings should be interpreted as AD-related differences in handwriting kinematics rather than as evidence of improved handwriting performance because handwriting quality was not assessed and path length was not normalized for numeral size. Nevertheless, quantitative kinematic assessment may provide objective information for evaluating handwriting ADs and for understanding how different device designs influence handwriting movement characteristics in children with disabilities.

## 1. Introduction

Handwriting is a fundamental means of communication and record-keeping that supports children’s participation and success in school and contributes to their self-esteem [1,2,3]. It requires precise control of the writing instrument through coordinated finger and hand movements, guided by haptic feedback and the integration of sensory, motor, and cognitive systems [4]. Children with neurological and motor disabilities may experience significant difficulty with handwriting due to abnormal muscle tone, muscle weakness, impaired coordination, and spasticity. These impairments can interfere with the ability to manipulate a writing instrument and regulate writing pressure [5,6,7]. As a result, children may demonstrate slow writing, irregular letter or numeral formation, reduced legibility, and increased fatigue during handwriting tasks [6,7]. Reduced handwriting ability may limit children’s expression of creativity, restrict their learning potential, and diminish their interest in educational activities [8]. Accordingly, interventions designed to improve handwriting performance are needed to support children’s participation in educational activities.

Handwriting assistive devices (ADs) may provide an alternative or complementary strategy for impaired hand function [9,10]. Various approaches have been developed to support handwriting, including grip-modifying devices, hand-support devices, customized writing aids, and mechanically stabilized devices [11,12,13,14]. These devices may reduce the demands placed on precise finger control, improve stabilization of the writing instrument, or compensate for limited grasp function. Previous studies have reported potential benefits in writing speed, accuracy, handwriting quality, pen control, and user comfort [11,12,13,14]. In a recent preliminary study, Lemire et al. [13] evaluated a mechanical handwriting AD and reported that children with mild motor difficulties were able to trace letters that they could not produce without the AD. However, handwriting ADs differ considerably in their design and support mechanisms, and these differences may influence how the writing instrument is controlled. Some devices primarily enlarge the grasping surface, allowing the user to hold the writing instrument with a broader grasp pattern, whereas others provide a support surface for the hand to reduce the need for sustained finger grasp. Therefore, the functional effect of a handwriting AD may depend not only on whether a device is used, but also on how the device supports grasp and writing instrument control.

In this context, spherical-grip and palmar-support ADs represent two distinct approaches to supporting handwriting. Both ADs secure the writing instrument within the device; however, they differ in how the user’s hand interacts with the device during handwriting. A spherical-grip AD provides a large rounded grasping surface, allowing the user to manipulate the writing instrument by actively grasping the device with a gross grasp pattern rather than relying primarily on the precise finger control required for conventional handwriting. In contrast, a palmar-support AD provides a broad surface that supports the hand while the device slides across the writing surface. The device is guided primarily by proximal upper-limb movement rather than by finger grasp, reducing the demand for active grasp and fine finger control. Thus, the primary distinction is that the spherical-grip AD retains active grasp through a modified grasping interface, whereas the palmar-support AD shifts control away from finger grasp toward proximal upper-limb movement. These distinct support mechanisms place different demands on gross grasp, fine finger control, and proximal upper-limb movement.

Although handwriting ADs may enhance handwriting performance, evidence regarding how these devices alter the underlying kinematics of handwriting remains limited [9,15]. Handwriting performance refers to functional outcomes of handwriting, such as legibility and accuracy. Handwriting kinematics refers to the movement characteristics underlying handwriting execution, including stroke characteristics, velocity, acceleration, path length, and pen pressure. Previous evaluations of handwriting ADs have often focused on task completion time, handwriting quality, template deviation, comfort, and user satisfaction [11,13,16]. These outcomes are clinically meaningful, but they do not fully characterize the movement processes underlying handwriting execution. Quantifying handwriting kinematics may therefore provide complementary information on how different handwriting AD designs influence handwriting control and may support more objective evaluation of handwriting ADs.

Traditional handwriting assessments have primarily focused on observable outcomes of the completed handwriting task, such as legibility, accuracy, template deviation, and completion time, which are commonly evaluated through visual inspection or manual measurement [9]. In contrast, computerized handwriting analysis enables continuous recording of pen-tip position, movement timing, and pressure using a digitizing tablet and digital pen. These recordings allow quantitative characterization of stroke-level and trajectory-based movement features that are not readily captured by traditional handwriting assessment [17,18]. Such analysis may provide complementary information on handwriting kinematics and help clarify how different handwriting AD support mechanisms affect handwriting execution and inform device evaluation [9].

Despite this potential, few studies have quantitatively examined how different handwriting AD support mechanisms influence handwriting kinematics in children with disabilities. In particular, it remains unclear whether the distinct control strategies provided by spherical-grip and palmar-support ADs are associated with different patterns of handwriting kinematics. Therefore, this preliminary study aimed to quantitatively compare handwriting kinematics during numeric copying among no-AD, spherical-grip AD, and palmar-support AD conditions in children with disabilities. We hypothesized that handwriting ADs would alter handwriting kinematics compared with no-AD and that these changes would differ between the spherical-grip and palmar-support ADs.

## 2. Materials and Methods

### 2.1. Participants

Children were recruited using a non-probability volunteer sampling through posters and announcements posted on the research bulletin board at the National Rehabilitation Center between December 2025 and May 2026. Seven children were assessed for eligibility, and all met the eligibility criteria and were enrolled in the study. The inclusion criteria were as follows: (1) age between 7 and 12 years; (2) a diagnosis of a neurological or motor disability affecting hand function; (3) difficulty grasping, stabilizing, or manipulating a conventional writing instrument; and (4) the ability to understand and perform the numeric-copying task. The exclusion criteria were as follows: (1) visual impairment that could interfere with viewing the presented numeric stimuli or performing the handwriting task; and (2) inability to understand the study instructions or perform the numeric-copying task. The study procedures were explained to all children in age-appropriate language, and written assent was obtained before participation. Written informed consent was also obtained from the participants’ parents or legal guardians. This study was approved by the Institutional Review Board of the National Rehabilitation Center (NRC-2025-02-007).

### 2.2. Measurements

Handwriting movements were recorded using a Wacom Intuos Pro Large digitizing tablet (active area: 37.7 cm × 25.3 cm) connected to a computer and a non-inking Wacom Pro Pen 3 (Wacom Co., Ltd., Saitama, Japan). Pen movements were recorded and processed using MovAlyzeR software (version 6.1; NeuroScript, LLC, Tempe, AZ, USA). Such tablet-based handwriting analysis has been widely used to objectively quantify spatial, temporal, pressure, and kinematic characteristics of handwriting across different populations [19,20,21]. The system recorded the horizontal and vertical coordinates of the pen tip and pen pressure at a sampling rate of 300 Hz. The same stylus and tablet calibration were used across all three conditions. Position data were low-pass filtered at 12 Hz to reduce noise [19]. The filtered data were segmented into successive strokes by identifying points on the handwriting trace corresponding to zero crossings of the vertical velocity profile. The measured handwriting variables included the number of strokes, writing duration, path length, average absolute velocity, peak vertical acceleration, straightness error, normalized jerk, pen pressure, and writing size. Based on previous quantitative handwriting studies, these variables were selected to characterize handwriting execution, including temporal, spatial, movement-control, and pen–surface interaction characteristics [9,22,23,24,25,26]. The number of strokes was defined as the number of movement segments identified based on zero crossings of vertical pen-tip velocity. Writing duration was defined as the elapsed time from the initial pen contact with the tablet to the final pen lift-off after completing the numeric response and was expressed in seconds (s). Path length represented the cumulative distance traveled by the pen tip and was expressed in centimeters (cm). Path length was not normalized for numeral size. Average absolute velocity represented the mean absolute velocity of pen-tip movement and was expressed in centimeters per second (cm/s). Peak vertical acceleration was calculated as the absolute magnitude of peak vertical acceleration for each stroke and was expressed in centimeters per second squared (cm/s^2^). Straightness error quantified the deviation of the stroke trajectory from a fitted straight line normalized by the distance between the stroke endpoints and was dimensionless [22,27]. Normalized jerk represented the change in acceleration during a single stroke and was normalized for stroke duration and size. It was calculated as $\sqrt{0.5\times \int j{\left(t\right)}^{2}dt\times T^{5}/L^{2}}$, where $j\left(t\right)$, T, and L represent jerk, stroke duration, and stroke length, respectively [28]. The resulting measure was unitless, with higher values indicating less smooth movement [23,24]. Pen pressure was calculated as the mean relative axial pressure during pen-contact periods and was expressed on a scale ranging from 0 to 8191 pen tablet units. Writing size was quantified from the sample-level x–y trajectory data and calculated as the product of the horizontal and vertical extents and was expressed in square centimeters (cm^2^). For each numeric response, horizontal extent was calculated as the difference between the maximum and minimum x coordinates, and vertical extent was calculated as the difference between the maximum and minimum y coordinates during pen-contact periods.

Writing duration, average absolute velocity, path length, and peak vertical acceleration were calculated across the complete writing trajectory, including both pen-down and pen-up movements. Pauses occurring within the trial were included in the calculation of writing duration. Straightness error, normalized jerk, pen pressure, and writing size were quantified using pen-down data.

### 2.3. Handwriting Assistive Devices

Two commercially available handwriting assistive devices (ADs) with different support mechanisms were used in this study: a spherical-grip AD and a palmar-support AD.

The spherical-grip AD (Arthwriter Hand Aid; Maddak, Inc., Wayne, NJ, USA) consisted of a large spherical gripping surface approximately 7.6 cm in diameter and weighed approximately 57 g. A 1.9 cm central opening accommodated the writing instrument, which was secured using a side thumbscrew. The enlarged spherical surface allowed the participant to manipulate the writing instrument using a gross grasp rather than directly grasping the narrow shaft of the instrument.

The palmar-support AD (Writing-Bird; North Coast Medical, Inc., Morgan Hill, CA, USA) was made of frosted acrylic and measured approximately 18.3 × 7.1 × 5.6 cm, with a weight of approximately 204 g and a writing-instrument opening diameter of 3.0 cm. The device supported the hand while sliding across the writing surface and was guided primarily through proximal upper-limb movement, thereby reducing the need for active grasp and fine finger control during writing.

### 2.4. Procedure

A quasi-experimental within-subject repeated-measures design was used. Participants were seated comfortably in a chair, with a monitor and the digitizing tablet placed on a desk in front of them. They performed the handwriting task in their usual self-selected posture, with the chair and table heights and digitizing tablet position adjusted according to individual comfort and preference. The non-writing hand was placed on the table during the task, and writing was performed directly on the tablet surface. Handwriting was assessed using a numeric copying task. Considering the participants’ age and developmental level, only numeric copying was administered. Arabic numerals were selected because they are familiar and meaningful symbols for children and are considered suitable for assessing handwriting in young or beginning writers [25].

Each participant performed the numeric copying task under three conditions: without an AD (no-AD), with the spherical-grip AD, and with the palmar-support AD. No additional therapeutic intervention, including hand-targeted therapy, was provided during the experimental session. The order of the three experimental conditions was counterbalanced across participants using all six possible condition sequences, which were assigned using a computer-generated random sequence. Each sequence was used once, with one sequence repeated for the seventh participant. The study procedure for each condition consisted of three stages: familiarization, numeric-copying task, and rest. First, participants completed a 10 min familiarization period. In the no-AD condition, this period was used to familiarize participants with handwriting directly on the digitizing tablet. In the two AD conditions, the hand and device positions were first adjusted to ensure a comfortable writing posture, followed by familiarization with the corresponding device. Second, participants completed five numeric-copying trials using their usual writing hand under the assigned condition. Five two-digit numbers—19, 82, 37, 64, and 50—were presented on the monitor in 72-point font in a randomized order for each condition. Each target number remained visible on the monitor throughout the copying task. These five numbers were selected so that all Arabic numerals from 0 to 9 were represented exactly once across the stimuli. Participants were instructed to copy each presented number onto the center of the digitizing tablet within 1 min using a self-selected numeral size within the tablet area. Each two-digit number was considered a single trial. For each outcome measure, the five trial values were averaged within each participant and condition, and these means were used for statistical analysis. Finally, a 5 min rest period was provided between conditions. Fatigue and discomfort were monitored during the rest period.

All seven participants completed all three experimental conditions. All trials were completed within the 1 min time limit without interruption or termination. No instances of poor fitting, device rejection, adverse events, or device intolerance occurred during testing. Each participant received approximately USD 33 as compensation for participation.

### 2.5. Statistical Analysis

All statistical analyses were performed using SPSS Statistics version 25.0 (IBM Corp., Armonk, NY, USA). Descriptive data were summarized using the mean, standard deviation, median, and interquartile range. The Friedman test was used to compare outcome measures across the no-AD, spherical-grip AD, and palmar-support AD conditions. Given the small sample size, exact *p*-values were calculated for the Friedman tests. When a significant overall difference was identified, exact pairwise comparisons of Friedman rank sums were performed according to the method of Eisinga et al. [29], with Bonferroni adjustment for the three pairwise comparisons. For the principal pairwise comparisons, the probability of superiority for dependent samples (PS_dep_) was reported as a paired effect size [30], with exact 95% confidence intervals calculated using the Clopper–Pearson method [31]. Given the preliminary and exploratory nature of this study, no multiplicity adjustment was applied across the outcome-level Friedman tests. Statistical significance was set at *p* < 0.05.

## 3. Results

Table 1 presents the demographic and clinical characteristics of the participants. Seven children with disabilities participated in the study, including four boys and three girls. Their mean age was 9.86 years, with a range of 7–12 years. Of the seven participants, five were diagnosed with spastic cerebral palsy, one with degenerative cerebellar atrophy, and one with porencephaly.

**Figure 1:**
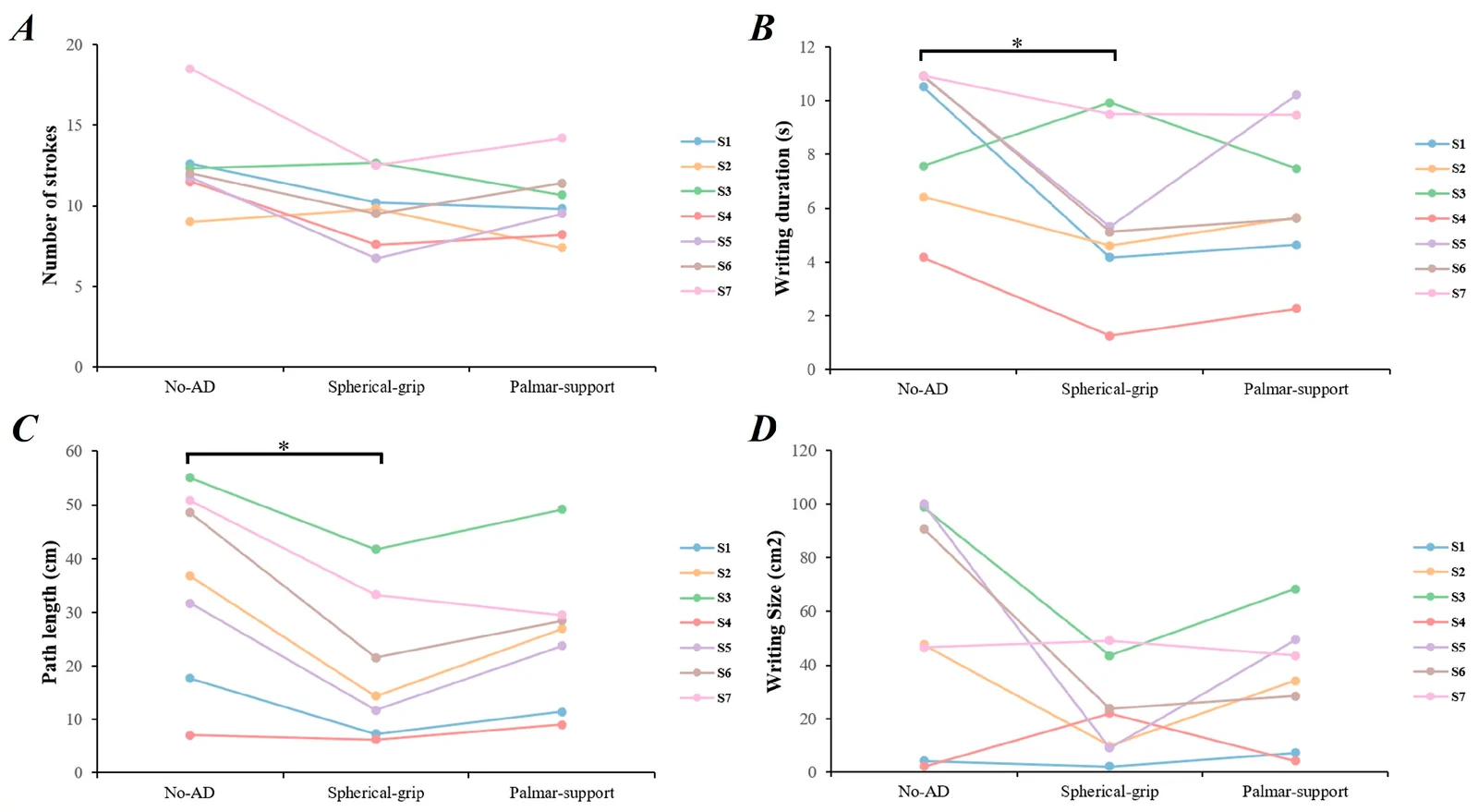
Individual participant responses across the three experimental conditions for (**A**) number of strokes, (**B**) writing duration, (**C**) path length, and (**D**) writing size. Each line represents an individual participant (S1–S7). An Asterisk (*) indicates significant pairwise differences between the no-AD and spherical-grip AD conditions after Bonferroni adjustment (writing duration, p_adj = 0.024; path length, p_adj = 0.002).

Table 2 presents the handwriting outcomes across the three experimental conditions. Regarding the handwriting outcomes, the Friedman test revealed significant differences in writing duration and path length among the three conditions (*p* < 0.05; Table 3). Post hoc pairwise comparisons with Bonferroni adjustment showed that writing duration and path length were significantly shorter in the spherical-grip AD condition than in the no-AD condition (*p* < 0.05). No significant differences were observed between the spherical-grip and palmar-support AD conditions or between the no-AD and palmar-support AD conditions (*p* > 0.05). There were also no significant differences among the three conditions in the number of strokes, peak vertical acceleration, straightness error, average absolute velocity, normalized jerk, pen pressure, or writing size (*p* > 0.05). Figure 1 presents the individual participant responses for the number of strokes, writing duration, path length, and writing size across the three experimental conditions. Individual participant responses for all nine measured handwriting outcomes are provided in Supplementary Figure S1.

## 4. Discussion

This preliminary study investigated the effects of spherical-grip and palmar-support handwriting ADs on handwriting kinematics in children with disabilities. The main findings were as follows: (1) writing duration and path length were significantly shorter in the spherical-grip AD condition than in the no-AD condition; (2) no statistically significant differences in writing duration and path length were observed between the spherical-grip and palmar-support AD conditions or between the palmar-support AD and no-AD conditions; and (3) the number of strokes, average absolute velocity, peak vertical acceleration, straightness error, normalized jerk, pen pressure, and writing size did not differ significantly among the three conditions. Accordingly, the study hypothesis was partially supported, as differences were detected between the spherical-grip AD and no-AD conditions for writing duration and path length, but not between the two AD conditions or between the palmar-support AD and no-AD conditions. Children with disabilities may experience difficulty stabilizing and manipulating a conventional pen because handwriting requires coordinated sensorimotor function and selective control of the hand and fingers [5,32]. For example, children with diplegic cerebral palsy showed lower prehension ability and greater variability in pen-grasp patterns than typically developing children, suggesting that the acquisition of mature pen-grasp patterns may be delayed in this population [5]. However, the relationship between pen-grasp pattern and handwriting performance is not straightforward. Previous studies have reported that pen-grasp pattern alone does not consistently determine handwriting speed or legibility in either children with cerebral palsy or typically developing children [33,34]. These findings suggest that providing a functionally suitable grasping interface may be more important than requiring a specific conventional pen grasp.

One possible mechanism underlying the shorter writing duration and path length in the spherical-grip condition may be related to the modified hand-pen interface. The spherical-grip AD enlarged the hand–pen interface and allowed the pen to be controlled through a gross, multidigit grasp. Previous grasp-synergy research has demonstrated that spherical grasping involves coordinated participation of multiple digits with relatively distributed force application across the fingers [35]. This broader grasping interface may have influenced grasp establishment and maintenance, potentially contributing to the shorter pen-tip path length and writing duration. Alternative explanations for the spherical-grip findings should also be considered. Both the number of strokes and writing size were numerically lower in the spherical-grip AD condition than in the no-AD condition. The numerically lower stroke count and writing size may have partly contributed to the shorter writing duration and path length observed in the spherical-grip condition. Thus, the observed differences in writing duration and path length should not be attributed solely to changes in the grasp interface.

For the palmar-support AD, no statistically significant differences in writing duration or path length were detected compared with the no-AD condition. Although this device reduced the need to grasp the pen directly, it required participants to guide the entire device across the writing surface using wrist and proximal upper-limb movements. Children with neurological and motor disabilities may have limitations in shoulder, elbow, and wrist control and reduced upper-limb strength [5], which could influence movement execution during a task requiring frequent directional changes.

No statistically significant differences were detected among the three conditions in the number of strokes, average absolute velocity, peak vertical acceleration, straightness error, normalized jerk, pen pressure, or writing size. Writing duration and path length are global outcome measures that reflect the total time and spatial extent required to complete a task, whereas variables such as normalized jerk and straightness error characterize the quality of movement execution within individual strokes. These results suggest that the spherical-grip AD was associated with differences in global temporal and spatial handwriting measures, while no statistically significant differences were detected in the other measured handwriting outcomes.

Normalized jerk has been widely used as an index of movement smoothness, with higher values indicating less smooth movement [24]. Previous studies suggested that jerk-related measures may be associated with handwriting performance. Toffoli et al. [26] reported associations between movement-smoothness measures, including logarithmic dimensionless jerk, and handwriting performance in school-age children. However, in the present study, no statistically significant difference in normalized jerk was detected across conditions. With respect to pen pressure, previous studies investigating the relationship between pen pressure and handwriting performance have reported inconsistent findings. For example, Coradinho et al. [36] reported a significant association between average pen pressure and handwriting quality in second-grade children, with better handwriting quality being associated with lower average pen pressure. However, Prunty et al. [37] found no significant association between pen pressure and handwriting legibility or speed in children with developmental coordination disorder. In addition, Lin et al. [38] observed that peak and average pen-tip force were not direct indicators of handwriting performance in typically developing children. These inconsistent findings suggest that the relationship between pen pressure and handwriting performance may vary depending on the population and handwriting outcomes examined. Given the small sample size and substantial inter-individual variability, these nonsignificant findings should not be interpreted as evidence that normalized jerk or pen pressure, or other handwriting outcomes were unaffected by the ADs. It remains uncertain whether these measures differ according to AD type and whether changes in these measures are associated with the observed differences in writing duration and path length. Future studies with larger samples should examine the effects of different AD types on these measures as well as the associations among handwriting variables.

This study has several limitations. First, the principal limitations of this study were the lack of handwriting quality assessment, including legibility, numeral completeness, and accuracy, and the lack of normalization of path length for numeral size. Therefore, the observed kinematic differences cannot be directly interpreted as improvements in the functional quality of handwriting. Second, the small sample size and heterogeneity in diagnosis, motor impairment, and age may have influenced interindividual variability in responses to the ADs and limited the statistical power, precision, and generalizability of the findings. In particular, differences in neurological diagnosis may be associated with distinct impairments in voluntary hand motor control, which may influence responses to the handwriting ADs. In addition, because multiple outcomes were examined without adjustment for multiplicity across outcomes, the findings for individual outcomes should be interpreted cautiously. Accordingly, the present results should be interpreted as preliminary and exploratory rather than as definitive evidence applicable to the broader population of children with disabilities. Third, this study investigated only the immediate effects of the ADs within a single assessment session following a brief familiarization period. Children may require longer practice to adapt their movement strategies, particularly when using an AD that substantially changes the grasp or support interface. Fourth, standardized hand-function and motor-severity measures were not collected, and relevant biomechanical factors, including grasp force, upper-extremity posture, and proximal joint movements, were not quantitatively assessed. Device-related mechanical factors, such as mass, friction, and device-tablet contact, were also not evaluated. Therefore, the potential contributions of these factors to the observed handwriting outcomes remain unclear. Despite these limitations, the present findings may provide reference data for future studies comparing different handwriting ADs and evaluating handwriting performance and kinematic outcomes such as writing duration and path length. Future studies should include larger samples, standardized hand-function assessments, and sufficient device familiarization, while evaluating longer-term effects using both kinematic and functional handwriting outcomes.

## 5. Conclusions

This preliminary study evaluated the effects of spherical-grip and palmar-support handwriting ADs on numeric handwriting kinematics in children with disabilities. The spherical-grip AD was associated with shorter writing duration and pen-tip path length compared with the no-AD condition, whereas no statistically significant differences were detected between the palmar-support AD and no-AD conditions in the measured handwriting outcomes. These findings should be interpreted as differences in handwriting kinematics rather than as evidence of improved handwriting performance because handwriting quality was not assessed and path length was not normalized for numeral size. The effects of handwriting ADs may vary according to device design. Quantitative kinematic assessment may nevertheless provide useful objective information for the evaluation of handwriting ADs.

## Figures and Tables

**Table 1:** Demographic characteristics of participants.

No	Age	Sex	Disorder	Affected Side	Dominant/Writing Hand	Prior Handwriting AD Use
1	11	F	Spastic cerebral palsy	Right	Left	None
2	12	F	Spastic cerebral palsy	Bilateral	Right	None
3	8	M	Degenerative cerebellar atrophy	Bilateral	Right	None
4	12	M	Spastic cerebral palsy	Left	Right	None
5	12	F	Spastic cerebral palsy	Bilateral	Right	None
6	7	M	Porencephaly	Bilateral	Left	None
7	7	M	Spastic cerebral palsy	Bilateral	Right	None

**Table 2:** Handwriting outcomes across the three experimental conditions.

	No-AD	Spherical-Grip AD	Palmar-Support AD
Number of strokes	12.53 ± 2.89 12.00 [11.50–12.60]	9.86 ± 2.23 9.80 [7.60–12.50]	10.17 ± 2.24 9.80 [8.20–11.40]
Writing duration (s)	8.77 ± 2.74 10.51 [6.42–10.92]	5.69 ± 3.06 5.11 [4.16–9.50]	6.47 ± 2.78 5.64 [4.63–9.46]
Peak vertical acceleration (cm/s^2^)	184.96 ± 240.12 91.87 [63.71–166.29]	115.26 ± 61.20 95.85 [56.47–190.09]	492.27 ± 565.44 295.15 [70.40–1070.59]
Straightness error	0.06 ± 0.01 0.057 [0.046–0.062]	0.06 ± 0.01 0.058 [0.056–0.067]	0.06 ± 0.01 0.060 [0.056–0.066]
Average absolute velocity (cm/s)	16.52 ± 12.31 17.07 [6.61–31.40]	6.33 ± 5.13 4.28 [2.91–12.36]	6.59 ± 4.43 4.95 [3.81–7.11]
Path length (cm)	35.36 ± 17.93 36.74 [17.71–50.84]	19.46 ± 13.53 14.41 [7.28–33.25]	25.45 ± 13.27 26.94 [11.40–29.50]
Normalized jerk	10,310.95 ± 13,089.25 936.01 [287.61–20,454.68]	1265.00 ± 1779.13 242.85 [130.08–1765.66]	5464.46 ± 13,563.78 230.90 [167.35–919.60]
Pen pressure (tablet units)	2874.08 ± 672.19 3238.44 [2378.65–3434.22]	2664.07 ± 724.12 2804.23 [1935.21–3362.96]	2535.14 ± 804.95 2812.69 [1789.57–3167.44]
Writing size (cm^2^)	55.70 ± 42.25 47.69 [4.17–98.73]	22.77 ± 17.90 21.91 [9.02–43.61]	33.58 ± 22.95 34.12 [7.08–49.47]

Values are presented as mean ± standard deviation and median [25th–75th percentile]. AD, assistive device.

**Table 3:** Statistical comparisons of handwriting outcomes across the three experimental conditions.

	χ^2^	df	Exact *p*	Kendall’s W	Post Hoc (p_adj)	Effect Size (PS_dep_)	95% CI
Number of strokes	5.429	2	0.085	0.388			
Writing duration (s)	8.000	2	0.016 *	0.571	b < a (0.024 *)	0.857	[0.421, 0.996]
Peak vertical acceleration (cm/s^2^)	3.714	2	0.192	0.265			
Straightness error	0.222	2	0.928	0.016			
Average absolute velocity (cm/s)	3.429	2	0.237	0.245			
Path length (cm)	10.286	2	0.004 *	0.735	b < a (0.002 *)	1.000	[0.590, 1.000]
Normalized jerk	2.000	2	0.486	0.143			
Pen pressure (tablet units)	2.000	2	0.486	0.143			
Writing size (cm^2^)	2.571	2	0.305	0.180			

χ^2^, Friedman test statistic; df, degrees of freedom; W, Kendall’s coefficient of concordance; PS_dep_, probability of superiority for dependent samples; CI, confidence interval. Exact *p*-values are reported for the omnibus Friedman tests. Significant omnibus results were followed by exact pairwise comparisons of Friedman rank sums according to Eisinga et al., with Bonferroni adjustment for three pairwise comparisons; p_adj denotes the adjusted exact *p*-value. PS_dep_ with exact 95% Clopper–Pearson confidence intervals was reported as the paired effect-size estimate. a, no-AD; b, spherical-grip AD. * *p* < 0.05.

## Data Availability

The original contributions presented in the study are included in the article, further inquiries can be directed to the corresponding author.

## Supplementary Materials

The following supporting information can be downloaded at: https://www.mdpi.com/article/10.3390/children13101385/s1, Figure S1: Individual participant responses across the three experimental conditions for all measured handwriting outcomes: number of strokes, writing duration, peak vertical acceleration, straightness error, average absolute velocity, path length, normalized jerk, pen pressure, and writing size. Each line represents an individual participant (S1–S7). Asterisks indicate significant pairwise differences between the no-AD and spherical-grip AD conditions after Bonferroni adjustment (writing duration, p_adj = 0.024; path length, p_adj = 0.002).
