# Supplementary material for: A Preliminary Study of the Immediate Effects of Spherical-Grip and Palmar-Support Handwriting Assistive Devices on Handwriting Kinematics During Numeric Copying in Children with Disabilities
Source: Children (Basel). 2026 Oct 12;13(10):1385. doi: 10.3390/children13101385 (PMC13703022; doi:10.3390/children13101385)
Supplement: Supplementary file 1 [file children-13-01385-s001.zip › children-4494400-supplementary.pdf]

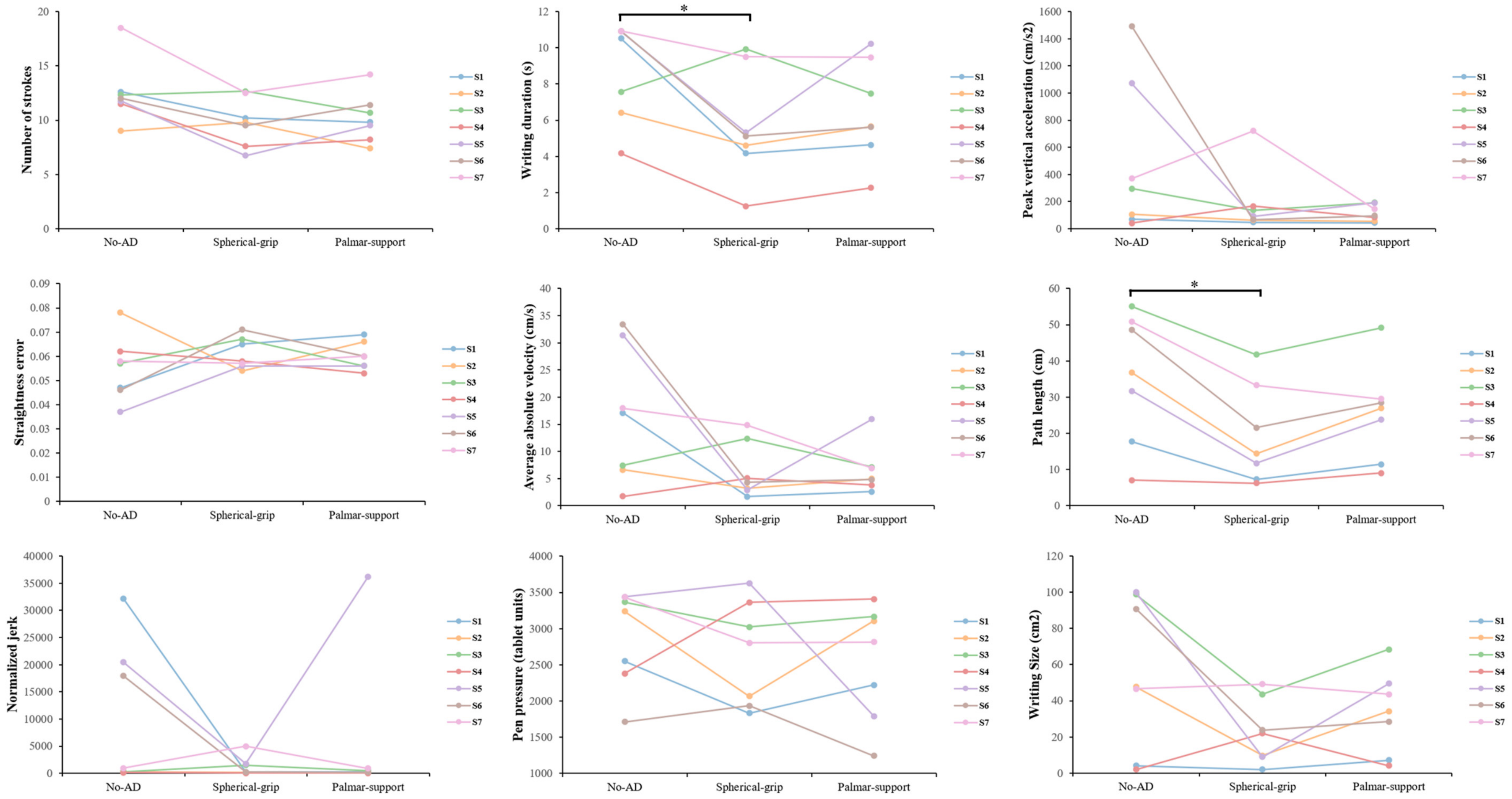

Supplementary Figure S1. Individual participant responses across the three experimental conditions for all measured handwriting outcomes: number of strokes, writing duration, peak vertical acceleration, straightness error, average absolute velocity, path length, normalized jerk, pen pressure, and writing size. Each line represents an individual participant (S1–S7). Asterisks indicate significant pairwise differences between the no-AD and spherical-grip AD conditions after Bonferroni adjustment (writing duration, p_adj = 0.024; path length, p_adj = 0.002).
